# The Indiana State Dental Association

**Published:** 1891-09

**Authors:** 


### The Indiana State Dental Association.

The thirty-third annual meeting of this society was held in the Dental College, at Indianapolis, on the 30th of June, continuing the sessions for three days.

This is one of the oldest of our State dental societies, and from what we saw during a visit to that meeting, and from the transactions which came to hand a few days ago, it is plain that this body is not suffering from age, it is, as we think clearly shown in its proceedings, gaining strength and vigor as it grows older. It is certainly keeping up in association work. Quite a number of very good papers were read, there were ten in all, and all equal to if not above the ordinary papers read before such bodies; they all deserve to be read, re-read, and studied.

One that attracted very general attention as presenting in a very happy manner the subject, was by Dr. M. F. Ault, entitled "An Ideal College Curriculum." This subject was handled with a master hand; the subject was so fully presented, and so well discussed, that it seemed a work of supererogation to add much in the way of discussion, still it was discussed by quite a number.

Two forenoons were devoted to clinics, in which many new ideas were presented, and here, as elsewhere, well conducted clinics elicited much and earnest attention, which will always be the case when they are properly conducted, and subjects of interest are presented in them.

The society is abreast with the foremost in its organization. Its business affairs are conducted without consuming much of the time of the general meeting, being almost exclusively in the hands of the Executive Committee. The promptness with which the proceedings of the association have been issued is very commendable indeed; this, however, is due to the fact that the volume is published by a lady, Mrs. W. M. Herriott, the Proprietress of the "Indianapolis Dental Depot"; and here, did time and space permit, we should be glad to specify what has been done by the energy, pluck and perseverance of this woman. Her husband, Dr. W. M. Herriott, many years ago established "The Indianapolis Dental Depot," this he did under some embarrassing circumstances, that it is not necessary here to mention, under the great efforts to which he was subjected his health gave way, and seven years ago he passed away. Mrs. Herriott, instead of letting the business run down, or pass into other hands, took personal supervision of it, and has had its sole management from that to the present time, working out a greater success, if possible, than her husband had done. Under her management the business has been better than ever before, has been much extended, and is conducted as systematically and by as good business methods as any such establishment in the country. The former embarrassment that rested upon it has, under her hand, entirely disappeared, and the whole enterprise is now a most successful and desirable one. This we regard as a heroic effort on the part of Mrs. Herriott, and one that will stand as an illustration of what an earnest, capable woman can do. It is gratifying to state that the dental profession of Indiana, and even of its vicinity, have nobly stood by "The Indianapolis Dental Depot," and we are sure that the best wishes of the profession will be extended for the success of Mrs. W. M. Herriott.

